# *Lactiplantibacillus pentoses* CCFM1227 Produces Desaminotyrosine to Protect against Influenza Virus H1N1 Infection through the Type I Interferon in Mice

**DOI:** 10.3390/nu15163659

**Published:** 2023-08-21

**Authors:** Qianwen Wang, Zhifeng Fang, Yue Xiao, Hongchao Wang, Pinghu Zhang, Wenwei Lu, Hao Zhang, Xiuwen Zhou

**Affiliations:** 1 State Key Laboratory of Food Science and Technology, Jiangnan University, Wuxi 214122, China; 13423674542@163.com (Q.W.); zhifengf@jiangnan.edu.cn (Z.F.); xiaoyue@jiangnan.edu.cn (Y.X.); luwenwei@jiangnan.edu.cn (W.L.); zhanghao61@jiangnan.edu.cn (H.Z.); 2 School of Food Science and Technology, Jiangnan University, Wuxi 214122, China; 3 Institute of Translational Medicine, Medical College, Yangzhou University, Yangzhou 225009, China; zhangpinghu@yzu.edu.cn; 4 Jiangsu Key Laboratory of Integrated Traditional Chinese and Western Medicine for Prevention and Treatment of Senile Diseases, Medical College, Yangzhou University, Yangzhou 225009, China; 5 National Engineering Research Center for Functional Food, Jiangnan University, Wuxi 214122, China; 6 Yangzhou Institute of Food Biotechnology, Jiangnan University, Yangzhou 225004, China; 7 Institute for Fetology, The First Affiliated Hospital of Soochow University, Suzhou 215021, China

**Keywords:** *Lactiplantibacillus pentosus*, influenza virus, antiviral probiotic, type I interferon, desaminotyrosine

## Abstract

Microbiota-derived desaminotyrosine (DAT) protects the host from influenza by modulating the type I interferon (IFN) response. The aim of this study was to investigate the antivirus effects of a DAT-producing bacteria strain. A comparative genomics analysis and UHPLC Q-Exactive MS were used to search for potential strains and confirm their ability to produce DAT, respectively. The anti-influenza functions of the DAT producer were evaluated using an antibiotic-treated mouse model by orally administering the specific strain before viral infection. The results showed the *Lactiplantibacillus pentosus* CCFM1227 contained the *phy* gene and produced DAT by degrading phloretin. In vivo, *L. pentosus* CCFM1227 re-inoculation increased the DAT level in feces, and protected from influenza through inhibiting viral replication and alleviating lung immunopathology. Furthermore, CCFM1227-derived DAT was positively correlated with the IFN-β level in the lung. The transcriptome results showed that CCFM1227 activated gene expression in the context of the defense response to the virus, and the response to interferon-beta. Moreover, CCFM1227 treatment upregulated the expression of MHC-I family genes, which regulate the adaptive immune response. In conclusion, *L. pentosus* CCFM1227 exerted antiviral effects by producing DAT in the gut, and this may provide a potential solution for creating effective antiviral probiotics.

## 1. Introduction

Although vaccination programs and antiviral drug treatments are widely applied, influenza virus infections still cause significant morbidity and mortality worldwide [1,2]. A previous study estimated that more than 290,000 seasonal influenza-associated respiratory deaths occur annually; people older than 75 and younger than 5 years are susceptible to influenza [3]. The innate immune system is the first line of immune defense against viral infection. However, respiratory RNA viruses, including influenza virus, respiratory syncytial virus (RSV), coronaviruses, and rhinoviruses, can suppress innate immune responses to provide an opportunity for efficient virus replication and further infection [4]. As the first line of defense against viral infection, the type I interferon (IFN) response plays a critical role. Type I IFNs induce the expression of antiviral proteins, inhibiting the virus replication directly [5]. Type I IFN can also cause an upregulation of major histocompatibility complexes (MHC), which are important for recognizing pathogens and can induce innate and adaptive immune responses [6,7,8]. In contrast, the influenza virus could inhibit IFN function via multiple pathways. For example, NS1, the non-structural protein of the influenza virus, contributes to attenuating the IFN response during infection. It appears to be the most crucial IFN-antagonist protein. Moreover, PB1-F2 protein, M2 protein, viral polymerase, and cellular inhibitory factors also suppress the type I IFN response [9]. Due to immune evasion, prophylactic vaccines or effective antiviral treatments against influenza virus infection are either unavailable or provide limited protection. Therefore, a practical approach is needed to regulate host immunity and resistance to influenza virus infection.

Intestinal microbes and metabolites play an essential role in the development and functioning of the host immune system. Abundant evidence has indicated crucial communication between the intestinal microbiota and the lungs [10]. Antibiotic (Abx) administration impairs innate and adaptive antiviral immune responses, as well as viral clearance, including type I IFN response [11]. Interestingly, gut microbiota-derived metabolite supplements could enhance the type I IFN response in the host during pathogenic infection. Recently, studies have confirmed that short-chain fatty acids, valine, coenzyme A, deoxycholic acid, and desaminotyrosine (DAT), could activate type I IFN to protect against viral infection [12,13,14,15]. Particularly, DAT, produced by a specific human-associated gut microbe, *Clostridium orbiscindens* (modified as *Flavonifractor (F.) plautii*) [16], rescued influenza virus-infected mice by priming the amplification loop of type I IFN signaling [13]. In addition, DAT was identified as an anti-inflammatory metabolite that modulates local and systemic immunity [17]. However, a high dose of DAT exhibited acute and sub-chronic oral toxicities [18]. Furthermore, *F. plautii*, considered a potential pathogen, is impossible to use as a probiotic [19]. Consequently, finding a safe and eatable bacterial strain that can produce DAT to prevent influenza virus infection makes practical sense. Given that phloretin hydrolase in *F. plautii* is coded by the *phy* gene and degrades phloretin to produce DAT [20], it is assumed that the bacterial strain containing the *phy* gene is a potential DAT producer, and might exert antiviral effects on influenza virus infection by regulating the immune response, particularly activating type I IFN.

In this study, we found the genome of *Lactiplantibacillus pentosus* CCFM1227 but not HY311 could be matched to the *phy* gene of *F. plautii*. Therefore, the ability to produce DAT in vitro and vivo was detected. In an antibiotic cocktail-treated mice model reinoculated by CCFM1227, the prophylactic effect of these strains on influenza virus infection was evaluated through analyzing inflammatory cytokines, lung injury parameters and architecture, and replication of the virus. Based on the fact that DAT could activate type I IFN response, the effects of special strain treatment on levels of IFN-β and induced antiviral-related proteins were examined. Finally, transcriptome analysis was used to investigate the other functions of the anti-influenza probiotic in the lung. Therefore, this study aimed to find an effective probiotic to protect against influenza virus infection, and explore the potential antiviral mechanisms of the probiotic.

## 2. Materials and Methods

### 2.1. Bacterial Strains and Culture Conditions

*Lactiplantibacillus (L.) pentosus* CCFM1227 and *L. pentosus* HY311 were stored at the Culture Collection of Food Microbiology (CCFM) in Jiangnan University. The strains were cultured in De Man, Rogosa, and Sharpe medium (MRS) containing 0.05% (*w*/*v*) L-cysteine-HCl at 37 °C for 12 h under anaerobic condition (AW500SG, Electrotek Scientific Ltd., Keighley, UK).

### 2.2. Identification of Candidate Genes

The amino acid sequences of phloretin hydrolase were downloaded from Uniprot (https://www.uniprot.org/, accessed on 5 March 2021) as a reference protein set. Orthologous proteins were identified using BLASTP with the following thresholds: E-value < 1 × 10^−10^, 30% identity. The Uniprot accession numbers of matched amino acid sequences included A0A2K9HZZ1, A0A1C1ZZD8, A0A3B8E9Y8, A0A3B8ERZ6, A0A494SAG9, A0A2S3U3Z7, A0A2S9WAP7, A0A2S9VJH5, A0A2S9VYT1, I9AIY1, and A0A3M6LPM8.

### 2.3. Flavonoid Degradation and DAT Production In Vitro

To determine the ability of bacterial strains to degrade flavonoids, a previously published protocol [13] was followed, with a modification. *L. pentosus* CCFM1227 or *L. pentosus* HY311 (2.5 × 10^8^ CFU) was incubated into MRS with and without 1.5% (*w*/*v*) phloretin for 24 h. An aliquot of the supernatant was blended with an equal volume solution of 2% (*w*/*v*) AlCl_3_ dissolved in methanol for 10 min to enhance color. Absorbance was measured under 415 nm using a universal microplate reader.

The ability of bacteria strains to produce DAT was determined, following a previously published protocol with a modification [21]. Briefly, *L. pentosus* CCFM1227 or *L. pentosus* HY311 was incubated at 37 °C in 5 mL MRS broth and grown for 12 h. The bacterial cells were harvested using centrifugation at 2400× *g* for 20 min at 4 °C, and then washed with PBS under the same centrifugation conditions. The cells were added into 1 mL M9 medium (2 mM MgSO_4_, 0.1 mM CaCl_2_, 33.7 mM Na_2_HPO_4_, 22 mM KH_2_PO_4_, 8.55 mM NaCl, 9.35 mM NH_4_Cl) supplemented with 0.5 mM phloretin and grown at 37 °C. The supernatant samples were collected at 12 h and 24 h, respectively. The supernatant was shaken at room temperature using a vortex machine for 1 min. To generate the crude methanol extract, 800 μL pre-cool methanol was added to 200 μL supernatant, and the mixture was blended by shaking. Then, the blend was placed on ice for 30 min and spun down at 15,000× *g* for 10 min at 4 °C to eliminate the proteins. The resulting methanol extract was dried under a vacuum at 45 °C for 3 h. Finally, the pellets were resuspended in 100 μL of methanol–water solution (*v*:*v* = 1:1). The separation was carried out on an ACQUITY UPLC BEH C18 column (Waters, Milford, Massachusetts; 1.7 μm, 2.1 × 100 mm), and the mass spectra were acquired using a Vanquish UHPLC Q-Exactive plus mass spectrometer [22] (Thermo Fisher, San Jose, CA, USA). All experiments were carried out in quadruplicate at each time point.

### 2.4. In Vivo Treatment of Mice

All experimental procedures were approved by the Ethics Committee of Yangzhou University (Approval No. 202201004). Briefly, 3–4-week-old ICR female mice (Charles River Co., Ltd., Beijing, China) were fed a standard chow diet and water ad libitum under controlled conditions with a 12 h light–dark cycle, 25 ± 2 °C, and 50% humidity.

After acclimating for 7 days, mice were randomly assigned to four groups (n = 10 each). Then, mice were intragastrically administrated for 1 week with the antibiotic cocktail: ampicillin 1.5 mg/mouse/day, vancomycin 0.75 mg/mouse/day, neomycin 1.5 mg/mouse/day, and metronidazole 1.5 mg/mouse/day. Before influenza virus infection, Abx-treated mice were gavaged with 200 μL of bacterial suspension (2.5 × 10^8^ CFU) or an equal volume of sterile saline, daily, for 2 weeks before viral infection.

**Figure 1:**
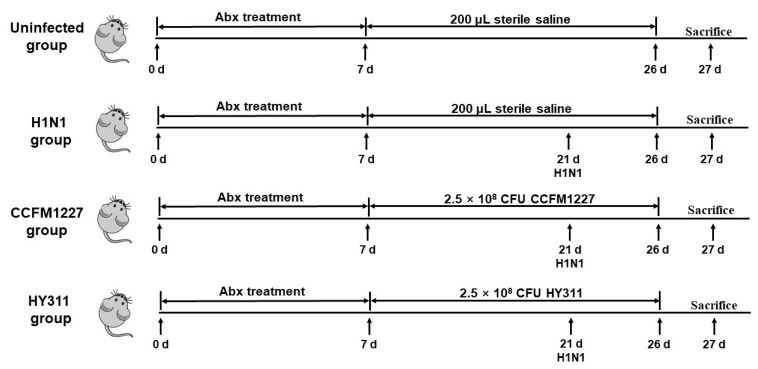
Timeline of mouse models.

For influenza virus infection, mice were injected intranasally with a dose of 50% lethal dose (LD_50_) of influenza A virus A/FM1/47 (H1N1), on day 21, provided by the Key Laboratory of Avian Infectious Diseases of Yangzhou University. The mice continued to be intragastric administered 200 μL of bacterial suspension or sterile saline during infection (Figure 1). The mice were weighed daily after H1N1 infection.

### 2.5. Quantification of DAT Concentrations in the Gut and Serum

Fecal samples were collected directly from the anus into sterile tubes on day 26. DAT in the feces was extracted with a 2:2:1 ratio pre-cooled methanol–acetonitrile–water mixture. Additionally, DAT in the serum was extracted with a 4:1 ratio pre-cooled methanol–water mixture. The DAT concentration was determined using UHPLC, which was described in Section 2.3.

### 2.6. Lung Injury Parameters

To obtain bronchoalveolar lavage fluid (BALF), lungs were lavaged three times with 800 μL phosphate-buffered saline. The protein content in BALF was measured using Enhanced BCA Protein Assay Kits (Beyotime Biotechnology Co., Ltd., Shanghai, China) to evaluate the permeability of the bronchoalveolar–capillarity barrier. The lactate dehydrogenase (LDH) activity in BALF was measured using LDH activity Assay Kits (Beijing Solarbio Science & Technology Co., Ltd., Beijing, China) to indicate general cytotoxicity.

### 2.7. Histological Analysis of Lung

The left lobes of the lungs were fixed in 4% paraformaldehyde. Embedded in paraffin blocks, lungs were sectioned at 5 μm sections and stained with hematoxylin and eosin (HE). The images were obtained using a Pannoramic MIDI digital scanner (3DHistech Ltd., Budapest, Hungary).

### 2.8. Viral Load and mRNA Expressions of Antiviral Proteins

The total RNA of lung tissues was extracted using a FastPure^®^ Cell/Tissue Total RNA Isolation Kit (Vazyme Biotech Co., Ltd., Nanjing, China) following the manufacturer’s instructions. First-strand cDNA was synthesized using HiScript^®^ III SuperMix for qPCR (Vazyme Biotech Co., Ltd., Nanjing, China), and stored at −20 °C. A real-time PCR reaction system was prepared using the iTaq Master SYBR Green Super Mix (Bio-Rad Laboratories Inc., Hercules, CA, USA) and carried out with a CFX96 Thermal Cycler (Bio-Rad Laboratories Inc.). The expressions of genes were calculated using the 2^−ΔΔCT^ method, and normalized to that of GAPDH. Table 1 shows the primer sequences.

### 2.9. Cytokine Quantification

Lung tissues were homogenized and lysed in RIPA buffer (200 μL/20 mg tissue; Beyotime Biotechnology Co., Ltd., Shanghai, China) containing a protease and phosphatase inhibitor cocktail (Beyotime Biotechnology Co., Ltd., Shanghai, China). The homogenates were clarified after centrifugation at 4 °C, and the following cytokine levels were quantified using commercial ELISA kits: tumor necrosis factor (TNF)-α, interleukin (IL)-6, IL-10 (R&D Systems, Minneapolis, MN, USA), and IFN-β (Senbeijia Co., Ltd., Nanjing, China).

The peripheral blood of mice was collected into EDTA anticoagulation tubes at room temperature. The serum was frozen at −20 °C. IFN-β was measured using a commercial ELISA kit (Senbeijia Co., Ltd., Nanjing, China).

### 2.10. RNA-Seq Analysis

For RNA-seq, the total RNA of lung tissue was extracted, and its quality assessed via agarose gel electrophoresis. All the RNA samples with a 260/280 ratio of around 2.0 were used for the cDNA libraries’ construction and sequenced by Majorbio Biotech. A differential expression analysis among the H1N1 group and *L. pentosus* CCFM1227 group (three samples from each group) was performed using the R (v.4.0.3) package DESeq2 (v.1.30.0) with the following thresholds: *p*-value < 0.05, fold changes > 2. Mouse gene sets for gene ontology analysis were downloaded from http://ge-lab.org/gskb/ accessed on 10 April 2022.

### 2.11. Statistical Analysis

Data were expressed as the mean ± standard error of the mean (SEM). A Kruskal–Wallis test (nonparametric) was performed to compare more than two groups with each other, followed by the two-stage step-up method post-test; statistical significance was considered at * *p* < 0.05, ** *p* < 0.01, *** *p* < 0.001, **** *p* < 0.0001. The data of Q-Exactive Plus MS analysis were integrated using Xcalibur software 2.0 version (Thermo Fisher Scientific Inc., Waltham, MA, USA). A correlation analysis was performed using Spearman’s rank correlation coefficient. For RNA-seq, the Wald test was applied to calculate *p* values. *p* < 0.05 was considered statistically significant.

## 3. Results

### 3.1. L. pentosus CCFM1227 Degrades Phloretin to Produce DAT

The amino acid sequences of phloretin hydrolase (*phy* gene) of *F. plautii* were downloaded from Uniprot as a query. Based on the bidirectional best hit (BBH) criterion, the hydrolase protein of bacterial strains was identified using BLASTP. The results exhibited that the genome of *L. pentosus* CCFM1227 showed 43% identity with the reference sequences, and *L. pentosus* HY311 did not contain the *phy* gene.

**Figure 2:**
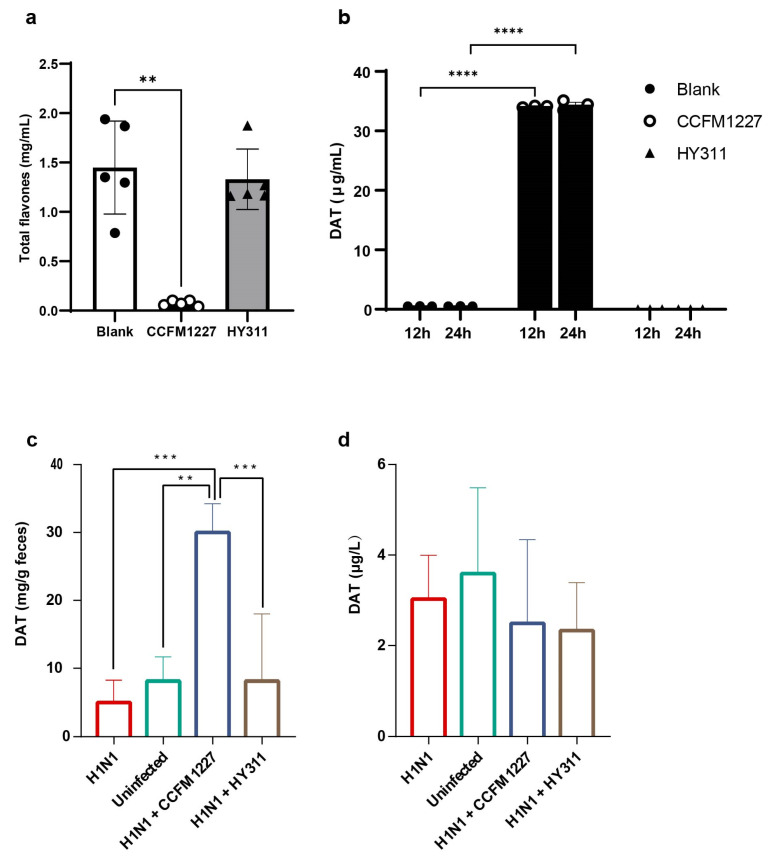
*L. pentosus* CCFM1227 degrades phloretin to produce DAT. (**a**) Phloretin degradation after incubation in darkness: *L. pentosus* CCFM1227 or *L. pentosus* HY311 (n = 5). (**b**) DAT quantification after 12 h and 24 h incubation in darkness: *L. pentosus* CCFM1227 or *L. pentosus* HY311 (n = 3). (**c**) Levels of DAT in the feces (n = 7). (**d**) Levels of DAT in the serum (n = 7). ** *p* < 0.01, *** *p* < 0.001, **** *p* < 0.0001.

*L. pentosus* CCFM1227, but not *L. pentosus* HY311, degraded the phloretin (Figure 2a). To confirm the product of phlorizin biotransformation, a Q-Exactive Plus MS analysis was applied to detect the levels of DAT in the supernatants. The results demonstrated that *L. pentosus* CCFM1227 could produce DAT, whereas *L. pentosus* HY311 did not transform phloretin to DAT (Figure 2b). Further, no significant alterations in DAT levels were observed in 24 h incubation, compared with 12 h incubation.

The DAT concentration in the feces and serum was quantified. The results indicated that the *L. pentosus* CCFM1227 treatment significantly increased the fecal DAT levels. In contrast, the *L. pentosus* HY311 did not induce an increase in the DAT levels in the guts (Figure 2c). However, both *L. pentosus* treatments did not affect the level of DAT in the serum samples (Figure 2d).

### 3.2. L. pentosus CCFM1227 Protects Mice against Influenza Virus Infection

**Figure 3:**
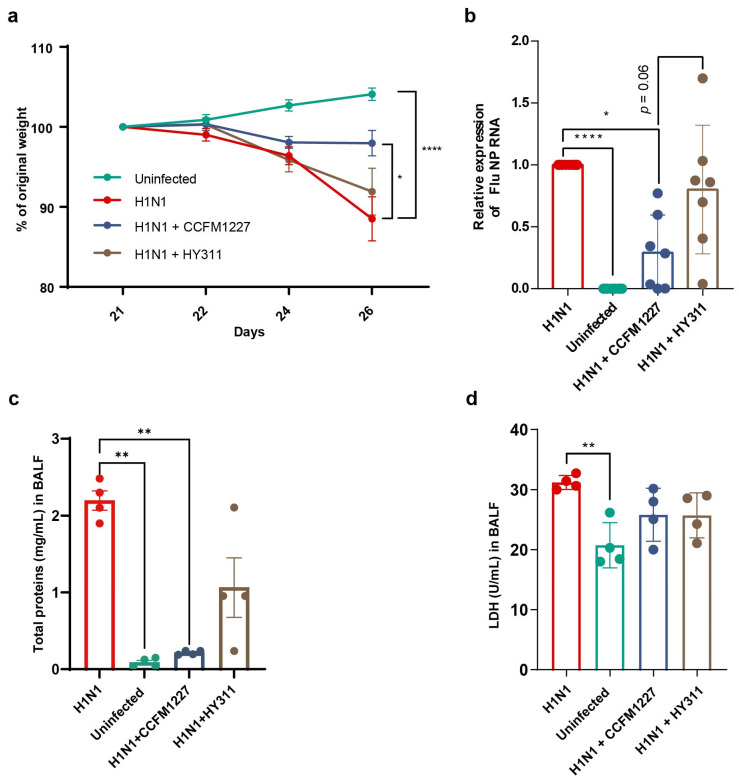
*L. pentosus* CCFM1227 protects against influenza in mice. (**a**) Percent weight loss from baseline after influenza virus infection (n = 10). (**b**) Viral load in lung tissues (n = 7–8) indicated by NP RNA expression. (**c**) Total protein concentration in BALF (n = 4). (**d**) Levels of the total activity of LDH in BALF (n = 4). * *p* < 0.05, ** *p* < 0.01, **** *p* < 0.0001.

To explore the antiviral effect of *L. pentosus* CCFM1227, the Abx-treated mice were orally administered with *L. pentosus* CCFM1227 or HY311, respectively, followed by influenza virus infection. Compared with the uninfected group, influenza virus infection induced a remarkable decrease in body weight, particularly from day 24 to day 26 (Figure 3a). There was no significant difference in body weight loss between infected mice from different groups before day 26 (Figure 3a). However, on day 26, the *L. pentosus* CCFM1227 but not HY311 significantly improved weight loss compared with the H1N1 group (Figure 3a). Moreover, the results of the relative expression of influenza virus NP RNA showed that CCFM1227 remarkably inhibited virus replication in the lungs. In contrast, the other *L. pentosus* strain did not reduce the viral load levels (Figure 3b).

The uninfected mice had low lung injury parameters (protein concentration and LDH activity in the BALF), indicating the integrality of the lung. However, increased protein content and LDH activity were found in BALF samples of the H1N1 group, indicating impairment of the alveolar–capillary barrier and lung cellular injury (Figure 3c,d). Orally administered CCFM1227 reduced the protein levels in BALF (Figure 3c). However, the results of LDH activity showed that general cytotoxicity in the lungs was not improved by the two bacterial strains (Figure 3d). These findings exhibited that *L. pentosus* CCFM1227, which produces DAT, could protect against influenza in vivo, including improving weight loss and inhibiting virus replication, as well as impairment of the alveolar-capillary barrier.

### 3.3. L. pentosus CCFM1227 Exerts Inhibitory Effects on Inflammation in the Lung

**Figure 4:**
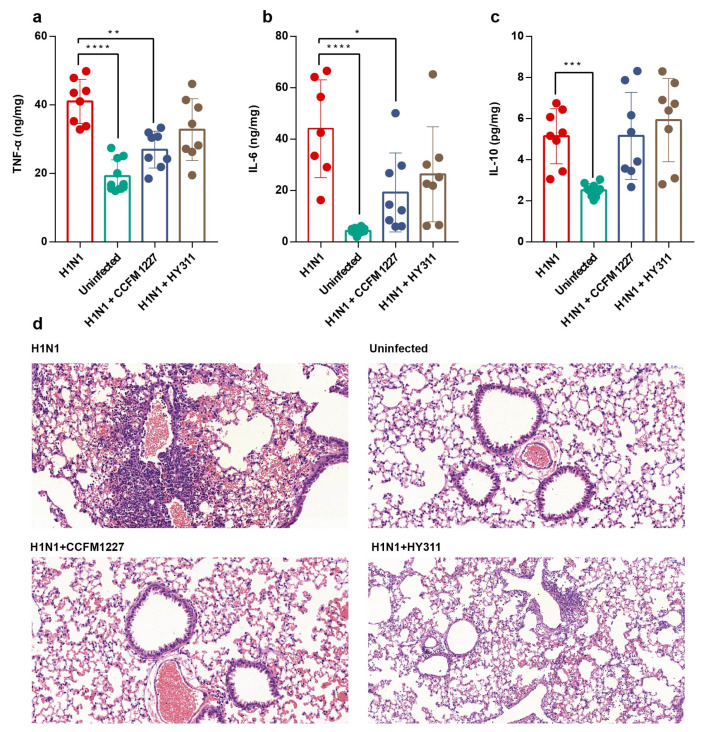
*L. pentosus* CCFM1227 decreased inflammation in the lung. Levels of (**a**) TNF-α, (**b**) IL-6, and (**c**) IL-10 in the lung tissue. (**d**) Lung sections stained with hematoxylin and eosin. Original magnification ×400. n = 8; * *p* < 0.05, ** *p* < 0.01, ***p < 0.001, **** *p* < 0.0001.

TNF-α, IL-6, and IL-10 are pro-inflammatory cytokines. It was shown that the levels of TNF-α and IL-6 in the lungs were significantly higher in H1N1 mice than that in uninfected mice (Figure 4a,b). *L. pentosus* CCFM1227 significantly downregulated the expressions of TNF-α and IL-6, while *L. pentosus* HY311 failed to reduce the levels of the two cytokines (Figure 4a,b). Compared with the uninfected group, the levels of IL-10 were significantly increased in the viral influenza infection group. However, the expression of IL-10 in *L. pentosus* treatments was comparable to that in the infection group (Figure 4c).

The histological structure is an important indicator for assessing inflammation in the lungs. Influenza virus infection induced pneumonia that destroyed the lung architecture. It caused perivascular and peribronchial infiltration of inflammatory cells, multiple focalized lesions, and hemorrhaging. The disappearance of nuclei from the bronchial epithelium and the shedding of the epithelium were observed. Besides, alveolar collapse and damage were found in the H1N1 group (Figure 4d). *L. pentosus* CCFM1227 treatment resulted in a diminution of pathological inflammation, particularly reduced infiltration of inflammatory cells. The alveolus and bronchial epithelium were not destroyed in the *L. pentosus* CCFM1227 group. However, the other *L. pentosus* strain HY311 was less effective in alleviating pathological inflammation compared to CCFM1227 (Figure 4d). Altogether, *L. pentosus* CCFM1227 treatment could partly reduce the levels of pro-inflammatory cytokines and alleviate inflammation in the lung.

### 3.4. L. pentosus CCFM1227 Augments Type I IFN Response in the Lung

**Figure 5:**
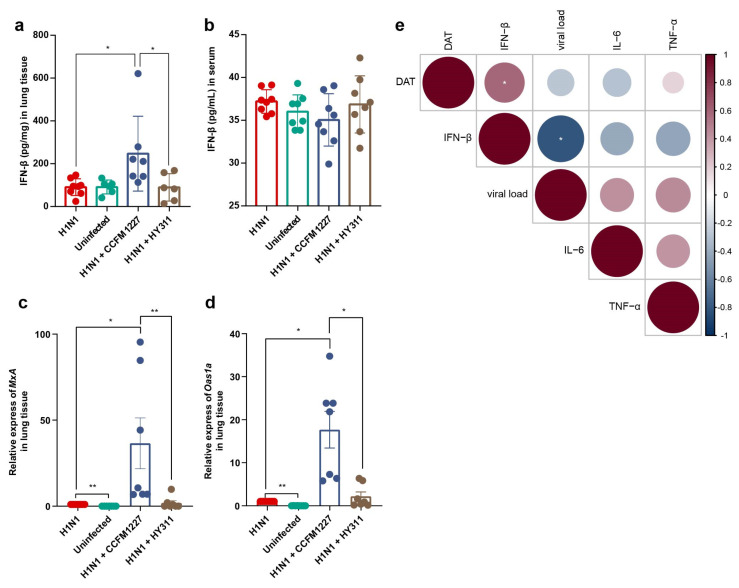
*L. pentosus* CCFM1227 actives type I IFN response in the lungs. (**a**) Levels of IFN-β in the lungs. (**b**) Levels of IFN-β in the serum. (**c**) Relative expression of *MxA* in the lungs. (**d**) Relative expression of *Oas1a* in the lungs. (**e**) Correlation analysis among gut DAT, lung cytokines, and viral load. n = 7; * *p* < 0.05, ** *p* < 0.01.

To estimate the type I IFN response activation, the expression of IFN-β (the major mediator of type I IFN response) and antiviral proteins were quantified. Influenza virus infection did not upregulate the expression of this cytokine in the lung. However, *L. pentosus* CCFM1227 treatment induced a significant increase in IFN-β levels in the lung (Figure 5a). In the serum, both influenza virus and *L. pentosus* strains did not alter the levels of IFN-β (Figure 5b). As type I IFN-induced human proteins, the mRNA expressions of myxovirus resistance protein A (*MxA*) and 2′-5′ oligoadenylate synthetase 1a (*Oas1a*) were used here to characterize the activation state of the type I IFN response. A significant elevation in the expression of *MxA* and *Oas1a* was observed in *L. pentosus* CCFM1227, but not the HY311 treatment group (Figure 5c,d).

Microbiota-derived DAT has been confirmed to protect against influenza virus infection through type I IFN. *L. pentosus* CCFM1227, a DAT-producer, might inhibit virus infection by increasing the levels of DAT in the gut. A correlation analysis was performed among the levels of DAT in the feces, cytokines in the lungs, and viral load, in order to determine the impact of DAT on suppressing virus infection. The results demonstrated that the DAT level in the feces was significantly positively correlated with the expression of IFN-β in the lung. Meanwhile, the level of IFN-β was significantly negatively correlated with the viral load in the lungs. However, there was no significant association between DAT levels and lung pro-inflammatory cytokines (IL-6 and TNF-α) (Figure 5e).

### 3.5. L. pentosus CCFM1227 Activates the Defensive Response to the Virus in the Lung Tissue

**Figure 6:**
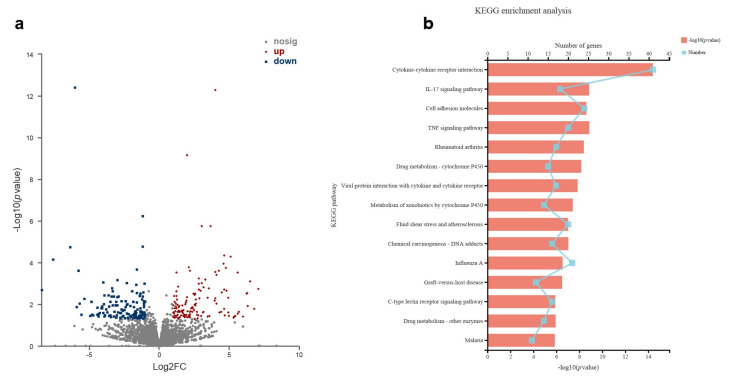

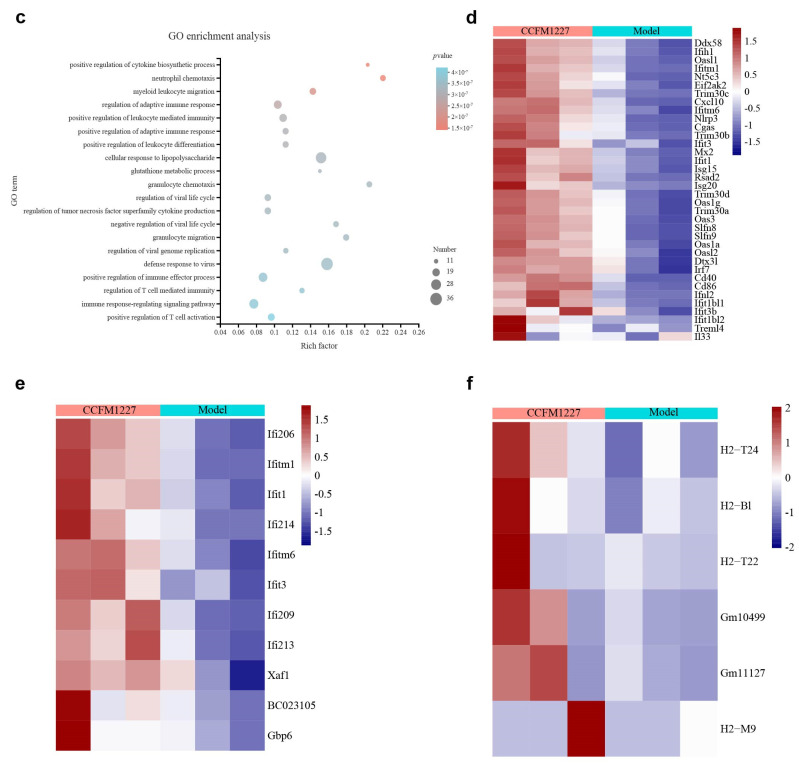
*L. pentosus* CCFM1227 affected the gene transcription profile in the lung. (**a**) Differentially expressed genes (DEGs) of the *L. pentosus* CCFM1227 group and H1N1 group. (**b**) KEGG enrichment analysis on DEGs. (**c**) GO enrichment analysis on DEGs. (**d**) Heat map representing the differently expressed genes that belong to the category of “the defensive response to virus” (GO: 0051607). (**e**) Heat map representing the differently expressed genes that belong to the category of the “response to interferon-beta” (GO: 0035456). (**f**) Heat map representing the differently expressed genes that are associated with MHC-I.

RNA-seq analysis was used to uncover more molecular pathways in the lung. *L. pentosus* CCFM1227 treatment significantly altered 849 genes’ expression levels in the lung, compared with the H1N1 group (Figure 6a). The Kyoto Encyclopedia of Genes and Genomes (KEGG) enrichment analysis on differentially expressed genes (DEGs) showed that *L. pentosus* CCFM1227 had enriched molecules in regulating immune responses, including “Cytokine–cytokine receptor interaction”, the “IL-17 signaling pathway”, the “TNF signaling pathway”, and “Viral protein interaction with cytokines and cytokine receptors”. CCFM1227 also altered the “influenza A” pathway and enhanced the expression of antiviral genes (Figure 6b). A gene ontology (GO) enrichment analysis revealed that among the DEGs, genes associated with “defense response to virus”, “negative regulation of viral life cycle”, and “regulation of viral genome replication” were highly enriched (Figure 6c). Most of the genes in the category of the defense response to virus (GO: 0051607) were significantly upregulated in *L. pentosus* CCFM1227-treated mice (Figure 6d). CCFM1227 increased gene expression in response to IFN-β (GO: 0035456) (Figure 6e). Excepted for interferons and antiviral proteins genes, *L. pentosus* CCFM1227 also upregulated the expression of *Cd40*, *Cd86,* and *Il33* (Figure 6d). Moreover, the DEGs enriched into categories related to leukocytes, including “myeloid leukocyte migration”, “positive regulation of leukocyte-mediated immunity”, “positive regulation of leukocyte differentiation”, “neutrophil chemotaxis”, “granulocyte chemotaxis”, and “granulocyte migration” (Figure 6c). *L. pentosus* CCFM1227 also modulated the “regulation of adaptive immune response” and the “positive regulation of adaptive immune response”. CCFM1227 treatment increased the expression of genes associated with MHC-I, including *H2-T22*, *H2-T24*, *H2-BI*, and *Gm11127* (Figure 6f). These results revealed that changes in the antiviral response, leukocyte functions, and adaptive immune responses were closely associated with *L. pentosus* CCFM1227 treatment in mice.

## 4. Discussion

Numerous studies indicate that gut microbiota and metabolites are vital for preventing respiratory diseases like viral and bacterial infections [10]. DAT, the degradation product of flavonoids, has been associated with improving influenza viral infection through type I IFN [13]. Therefore, the gut bacterium that produces DAT can potentially protect from influenza. DAT-producing probiotics among safe and eatable bacteria, such as *Bifidobacterium* and *Lactobacillus*, could contribute to the applications of these probiotics to the treatment of influenza virus infection. The present study aimed to find DAT-producing bacteria and explore their antiviral effects.

*F. plautii* produces DAT by degrading phloretin with phloretin hydrolase encoded by the *phy* gene [20]. The study found the *phy* gene in the genome of *L. pentosus* CCFM1227. Furthermore, the *L. pentosus* CCFM1227 was confirmed to degrade phloretin to produce DAT in vitro, whereas *L. pentosus* HY311 did not. Thus, it is strain-specific that *L. pentosus* CCFM1227 degrade phloretin. The Q-Exactive Plus MS analysis showed that the concentration of DAT did not reduce over time, which means that *L. pentosus* CCFM1227 did not degrade DAT, and DAT was the end product. We speculated that *L. pentosus* CCFM1227, as a DAT-producer, had the potential to protect the host against influenza.

To prove the above hypothesis, a mouse model of H1N1 infection was used to explore the antiviral effect of *L. pentosus* CCFM1227 and HY311 in the subsequent study. The results showed that *L. pentosus* CCFM1227 exerted prophylactic effects on the influenza virus infection. It could decrease viral load and weight loss, improve lung damage, and alleviate pathological lung symptoms. In contrast, *L. pentosus* HY311 did not increase resistance to influenza virus infection. Moreover, *L. pentosus* CCFM1227 uniquely modulated the inflammatory responses in the mice after influenza virus infection. Influenza virus infection causes excessively elevated levels of cytokines, known as cytokine storms [23], which lead to significant respiratory disease and widespread tissue damage [24]. IL-6 and TNF-α are typical pleiotropic cytokines produced in response to tissue damage and infection [25]. In this study, the results showed that *L. pentosus* CCFM1227 treatment decreased IL-6 and TNF-α levels in the lung tissue, as well as IL-6 in the blood (Figure S1a). Thus, *L. pentosus* CCFM1227 might suppress the cytokine storms to alleviate the inflammation and damage in the lung. Interestingly, influenza decreased the levels of TNF-α in the serum (Figure S1b). Influenza virus infection might result in depression or suppression of cellular responses to reduce TNF-α [26]. For example, influenza viruses fail to efficiently activate TNF-α gene expression in peripheral blood-derived DCs [27]. TNF-α has multiple functions, such as induction of adaptive immunity, cell apoptosis, proliferation, and inhibition of tumor cell growth [28]. *L. pentosus* CCFM1227 treatment restored the TNF-α in the serum, consistent with oseltamivir therapy [29]. Except for cytokines, *L. pentosus* CCFM1227 also affected the levels of blood immunocytes (Figure S1c–f), particularly platelets (Figure S1f). Platelets contain TLRs and TNF receptors, which are essential for activating immune responses [30]. The cross-cation of platelets and neutrophils tightly regulates host immune and complement responses as the initial intrinsic defense against influenza [31]. In addition, activated platelets could phagocytose viruses, generate reactive oxygen species, and activate other immune cells [32,33]. Patients with thrombocytopenia are more likely to have life-threatening infections with influenza virus, indicating an important role for platelets during virus infection [34]. Our results suggested that *L. pentosus* CCFM1227 treatment could ameliorate inflammation in the lung tissue. In the meantime, it might activate the platelets and TNF-α in the blood circulation. However, how the local and peripheric changes contribute to resistance to the virus still needs to be further investigated.

Steed et al. demonstrated that gut microbiota-derived DAT protects from influenza through type I IFN. Therefore, the antiviral mechanisms of *L. pentosus* CCFM1227 might be associated with the activation of type I IFNs, which are major antiviral effectors that mediate antiviral responses. The expression of IFN-β and antiviral proteins was evaluated to prove this hypothesis. In this research, H1N1 infection did not induce the type I IFN response, which is regarded to be effective against influenza viruses [5]. The previous report showed that the influenza virus has multiple mechanisms to suppress the type I IFN response, allowing for evasion of elimination [9,35]. Besides, Abx-treated mice exhibited delayed viral clearance due to severely diminished immune cell activation and impaired type I IFN production [36]. Notably, *L. pentosus* CCFM1227 treatment increased levels of IFN-β and antiviral proteins, while *L. pentosus* HY311 had fewer effects on this immune response. It was indicated that *L. pentosus* CCFM1227 could overcome the virus inhibitory mechanisms and activate the type I IFN pathway to protect against virus infection.

Previous studies have demonstrated that the gut microbiota is essential to activate type I IFN to defend against viral infection [36]. The gut can contribute to responses in the lungs in three main ways: (1) soluble microbial components and metabolites are spread through the bloodstream; (2) host-derived inflammatory mediators spill into the lung via the circulation; and (3) immune cells migrate from the intestine to the respiratory tract directly, via the circulation [10,37]. To explore the activation mechanisms of type I IFN via oral *L. pentosus* CCFM1227 administration, IFN-β levels were detected in the serum. The results showed that *L. pentosus* CCFM1227 did not alter the levels of IFN-β in the serum, which indicated that IFN-β did not directly transport from the immune organ via the blood system. Moreover, DAT levels were detected in the feces and the serum. Interestingly, *L. pentosus* CCFM1227 treatment significantly increased the production of DAT in the gut, and the levels of DAT were positively correlated with the expression of IFN-β in the lung. However, *L. pentosus* CCFM1227 did not alter the DAT levels in the serum. This finding means that this metabolite did not spill into the blood circulation and was not transported to the lung via circulation, so it might indirectly activate type I IFN in the lung. Thus, we speculated that *L. pentosus* CCFM1227 and its antiviral metabolite might exert antiviral effects by relying on immune cell migration. The migration of immune cells from the gut to the lung might be beneficial to enhance the function of the host to resist infection. For example, the migration of IL-22-producing ILC3s to the lung is needed for protection against pneumonia, which is initiated by sensing commensal bacteria via intestinal DCs [10]. Microbiota-derived SCFAs influence hematopoietic precursors in the bone marrow, which subsequently migrate to the lungs and shape an anti-inflammatory milieu [38]. Steed et al. found that lung phagocytes and bone-marrow-derived macrophages (BMDMs) in DAT treatment are essential mediators of type I IFN-mediated protection from influenza virus infection [13]. Colonization of *B. coccoides* in antibiotic-treated mice can also counteract the intrinsic defects in the expression of IFN-I and key ISGs in BMDMs [36].

According to transcriptome results, *L. pentosus* CCFM1227 noticeably activated the antiviral response to defend against influenza virus infection. Consistent with the above results, CCFM1227 activates the type I IFN response. Except for interferons and normal antiviral proteins, this bacterial strain also induced upregulated expression of *Il33*, which is inhibited by influenza virus infection. IL-33 promotes myeloid cell migration [39]. It might contribute to active leukocyte migration to the lung. Leukocytes can directly kill the virus or trigger humoral immunity to provide immune responses during viral infection [40]. The clinical outcomes showed that the downregulation of *IFITM1* leukocytes was a distinct characteristic of severe H1N1 disease [41]. *IFITM1* encodes interferon-induced transmembrane (IFITM) proteins which are crucial for protection against influenza. These findings indicated that *L. pentosus* CCFM1227 might activate the expression of *IFITM1* in leukocytes through IL-33. The functions of leukocytes and IL-33 require further research.

Moreover, the transcriptome results showed that CCFM1227 activated neutrophil chemotaxis, granulocyte chemotaxis, and granulocyte migration. Derived from hematopoietic stem cells, neutrophils carry antimicrobial peptides such as defensins and cathelicidins, which could neutralize the influenza A virus. Neutrophils also function as antigen-presenting cells (APCs) to guide CD8^+^ T cell activation and recruitment into the lungs [42]. Besides, the results indicated that *L. pentosus* CCFM1227 might enhance antigen processing and presentation to activate the adaptive immune response. It upregulated *H2-T22*, *H2-T24*, *H2-BI*, and *Gm11127*, belonging to the MHC-I family in the category of the adaptive immune response. Cytotoxic CD8^+^ T cells promote viral clearance during influenza viral infection by recognizing MHC-I-delivered viral peptides on the surface of infected cells to trigger the delivery of cytotoxic molecules and the release of antiviral cytokines to kill the infected cells [8]. Some MHC-II molecules also present peptides derived from extracellular pathogens to T cells, via endosomes, thereby generating an immune response against the pathogen [43]. Influenza virus might downregulate MHC-I surface expression levels to evade innate and adaptive immunity [6,8]. The lung tissue expressed higher levels of MHC, likely associated with the effects of type I IFN activated by *L. pentosus* CCFM1227 [7,44]. The various antiviral mechanisms of CCFM1227 require further investigation in the future.

In conclusion, *L. pentosus* CCFM1227 containing the *phy* gene is strain-specific in its production of DAT by degrading phloretin. In vivo, oral CCFM1227 was more effective in protecting against influenza A than the other *L. pentosus* strain, which does not produce DAT. The *L. pentosus* CCFM1227 treatment activated the type I IFN response by increasing DAT in the gut. Moreover, microbiota-derived DAT might exert antiviral effects, relying on immune cell migration. *L. pentosus* CCFM1227 also enhanced leukocyte functions and the host adaptive immune response, which might be essential to defend against influenza virus infection. The present study found that *L. pentosus* CCFM1227 had various mechanisms to prevent influenza, and these findings can contribute to the development of gut microbes as prophylactic agents for influenza virus infection.

## Figures and Tables

**Table 1:** Primer sequences for real-time PCR.

Gene	Forward/Reverse	Sequence (5′ to 3′)
GAPDH	Forward	AATGGTGAAGGTCGGTGTGAAC
	Reverse	GCCTTGACTGTGCCGTTGAA
NP	Forward	GGCACCAAACGGTCTTACGA
	Reverse	TCACCTGATCAACTCCATTACCA
MxA	Forward	CCAACTGGAATCCTCCTGGAA
	Reverse	GCCGCACCTTCTCCTCATAG
OAS1	Forward	GAAGAGGCTGATGTGTGGCT
	Reverse	TGTCCAGTTCTCTTCTACCTGC

## Data Availability

The data presented in this study are available on request from the corresponding author.

## Supplementary Materials

The following supporting information can be downloaded at: https://www.mdpi.com/article/10.3390/nu15163659/s1, Figure S1: *L. pentosus* CCFM1227 regulates levels of cytokines and blood cells.
