# Supplementary material for: Lactiplantibacillus pentoses CCFM1227 Produces Desaminotyrosine to Protect against Influenza Virus H1N1 Infection through the Type I Interferon in Mice
Source: Nutrients. 2023 Aug 21;15(16):3659. doi: 10.3390/nu15163659 (PMC10458433; doi:10.3390/nu15163659)
Supplement: Supplementary file 1 [file nutrients-15-03659-s001.zip › nutrients-2545356-supplementary.pdf]

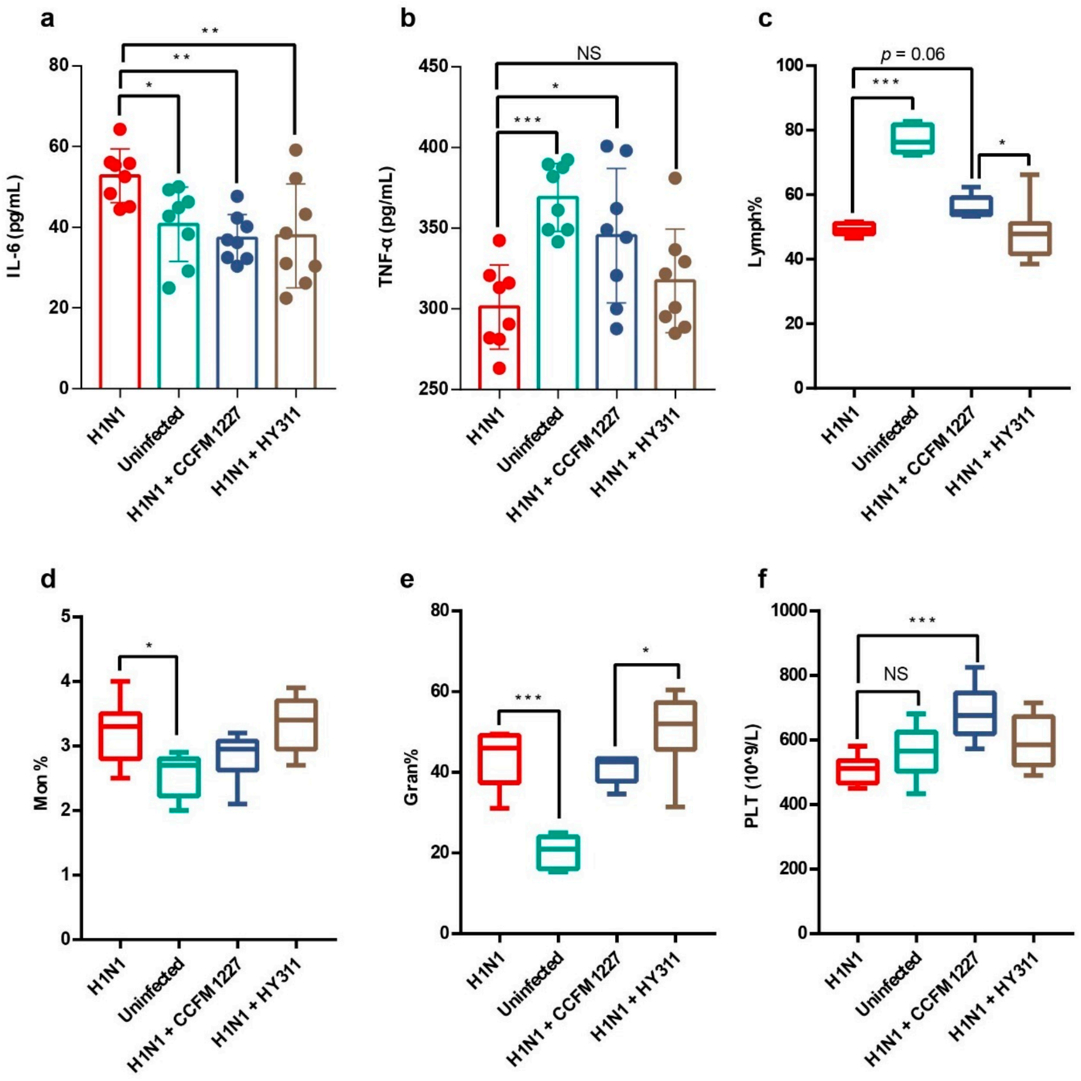

Figure S1. *L. pentosus* CCFM1227 regulates levels of cytokines and blood cells. Levels of (a) IL-6 and (b) TNF-α in the serum. Proportions of (c) lymphocytes (Lymph%), (d) monocytes (Mon%), and (e) granulocytes (Gran%). (f) Platelets (PLTs) in blood. n=8; *$p < 0.05$, **$p < 0.01$, ***$p < 0.001$, NS: no significance.
